# Boundary layer control by a fish: Unsteady laminar boundary layers of rainbow trout swimming in turbulent flows

**DOI:** 10.1242/bio.020008

**Published:** 2016-11-08

**Authors:** Kazutaka Yanase, Pentti Saarenrinne

**Affiliations:** Department of Mechanical Engineering and Industrial Systems, Tampere University of Technology Korkeakoulunkatu 6, Tampere FI-33101, Finland

**Keywords:** Particle image velocimetry, *Oncorhynchus mykiss*, Drag reduction, Swimming performance

## Abstract

The boundary layers of rainbow trout, *Oncorhynchus mykiss* [0.231±0.016 m total body length (*L*) (mean±s.d.); *N*=6], swimming at 1.6±0.09* L *s^−1^ (*N*=6) in an experimental flow channel (Reynolds number, *Re*=4×10^5^) with medium turbulence (5.6% intensity) were examined using the particle image velocimetry technique. The tangential flow velocity distributions in the pectoral and pelvic surface regions (arc length from the rostrum, *l_x_*=71±8 mm, *N*=3, and *l_x_*=110±13 mm, *N*=4, respectively) were approximated by a laminar boundary layer model, the Falkner−Skan equation. The flow regime over the pectoral and pelvic surfaces was regarded as a laminar flow, which could create less skin-friction drag than would be the case with turbulent flow. Flow separation was postponed until vortex shedding occurred over the posterior surface (*l_x_*=163±22 mm, *N*=3). The ratio of the body-wave velocity to the swimming speed was in the order of 1.2. This was consistent with the condition of the boundary layer laminarization that had been confirmed earlier using a mechanical model. These findings suggest an energy-efficient swimming strategy for rainbow trout in a turbulent environment.

## Abstract (precis)

**Summary:** The boundary layer laminarization of rainbow trout swimming in turbulent flows was confirmed. The results suggested an energy-efficient swimming strategy of this species in the turbulent flow environment.

## INTRODUCTION

The viscosity of water causes the flow close to the surface of any biotic or abiotic object to move more slowly (Schlichting, 1979). This spatial gradient in flow is known as the boundary layer. Skin frictional drag, which is the dominant factor in the total drag of a swimming fish, is created in the boundary layer due to the viscosity of the fluid (Webb, 1975). Therefore, the boundary layer is an important component of the hydrodynamics of fish swimming. Furthermore, the boundary layer over the surface of a fish's body plays a major role in determining the signals detected by a lateral line mechanoreceptor (reviewed by McHenry et al., 2008). Despite the critical roles of the boundary layer in swimming hydrodynamics and lateral line flow sensing in fishes, the boundary layer structure of a swimming fish has rarely been studied experimentally. The exceptions include Anderson et al. (2001) and the authors' previous measurements (Yanase and Saarenrinne, 2015). Experiments conducted on swimming fish by Anderson et al. (2001) investigated the boundary layer over the body surfaces of two marine species, the scup (*Stenotomus chrysops*; a carangiform swimmer) and the smooth dogfish (*Mustelus canis*; an anguilliform swimmer). The experiments revealed the oscillation between the laminar and turbulent boundary layers, which was in agreement with such known boundary layer models as the Blasius or Falkner−Skan equation for the laminar boundary layer, and the law of the wall for the turbulent boundary layer, respectively. In this regard, however, the surface motion phase-related characteristics of the unsteady boundary layer are not fully documented in Anderson et al. (2001). Therefore, there is still a lack of quantitative evidence to support the boundary layer laminarization over the undulatory fish surface.

An experiment using a mechanical model that emulated the undulatory motion of an aquatic animal had been performed previously by Taneda and Tomonari (1974). They demonstrated that the boundary layer over the surface of a motor-driven undulatory plate laminarized at the wave-crest when the ratio of the velocity (*c*) of the wave travelling downstream to the free stream velocity (*U*) was 1.2. Shen et al. (2003) confirmed these results numerically for turbulent flow over the surface of a smooth wavy wall that was undergoing transverse motion in the form of a stream-wise travelling wave with constant amplitude. At the *c*/*U* ratio of 1.2, the net power input that is required to counteract incoming flow was found to be at its minimum. Kunze and Brücker (2011) demonstrated that the tangential flow-velocity (*u*) distribution within the boundary layer over the surface of a fish-like moving plate with increasing amplitude downstream oscillated between a laminar and a turbulent flow profile throughout the cycle of undulatory motion at that *c*/*U* ratio.

Yanase and Saarenrinne (2015) have successfully measured the boundary layers of *Oncorhynchus mykiss*, a subcarangiform swimmer, swimming at a low swimming speed of 1.0* L *s^−1^ in a controlled experimental flow channel. This was the first time that the boundary layers of a swimming fish were measured using the particle image velocimetry (PIV) technique. PIV is an optical method of flow visualization that is based on the statistical correlation of small interrogation areas with high particle density. Thus, PIV offers significant advantages for the direct determination of the surface-normal gradient of longitudinal velocity in a highly heterogeneous flow field. Yanase and Saarenrinne (2015) observed that the velocity distribution within the boundary layers of rainbow trout swimming at 1.0* L* s^−1^ oscillated above and below the classical logarithmic law of the wall for a turbulent boundary layer with body motion (von Kármán, 1930). The logarithmic law of the wall is a self-similar solution for the mean velocity parallel to the wall (Schlichting, 1979).

Unlike the findings of Anderson et al. (2001), the boundary layers of rainbow trout swimming at 1.0* L* s^−1^ were found to be in a turbulent flow regime during the entire cycle of transverse surface motion (Yanase and Saarenrinne, 2015). Moreover, there was no sign of the Bone−Lighthill boundary layer thinning hypothesis (Lighthill, 1971), i.e. large drag augmentation resulting from a reduction in the boundary layer thickness due to the lateral movements of the body segments of swimming fish. However, it is not certain whether the boundary layer phenomena observed in Yanase and Saarenrinne (2015) are typical characteristics for a swimming rainbow trout over a range of sustained swimming speeds. Therefore, to clarify this uncertainty, the present study examined the boundary layer of rainbow trout that were swimming at a higher swimming speed of 1.6* L* s^−1^ in a 0.37 m s^−1^ free stream on the rationale that a 1.6* L* s^−1^ swimming speed may be energetically more efficient for rainbow trout than the 1.0* L* s^−1^ swimming speed in our previous experiment (Yanase and Saarenrinne, 2015). The Reynolds number (*Re*) of the experimental flow field used in the present study was 4×10^5^ based on the distance of the flow channel and freestream velocity. This suggests a transitional flow regime from laminar to turbulent [the critical values typically being *Re*=3.5–5.0 (×10^5^) (Schlichting, 1979)]. The 0.37 m s^−1^ freestream velocity was the maximum limit of the flow speed in our experimental flow system, where a two-dimensional PIV measurement was available with the desirable spatial resolution (8×8 or 6×6 pixels^2^ interrogation window size with 50% overlap) to determine the surface-normal gradients of the tangential flow velocity (*u*) in the boundary layer and, thus, the viscous stress
(1)
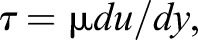
where μ is the dynamic viscosity of the fluid (Newton's law of friction). The hypothesis of the present study was that, if the boundary layer of rainbow trout could laminarize without separation, it would result in a substantial reduction in the skin friction in order for the fish to achieve a more energy-efficient swimming speed.

## RESULTS

### Boundary layer-related parameters

**Fig. 1.**
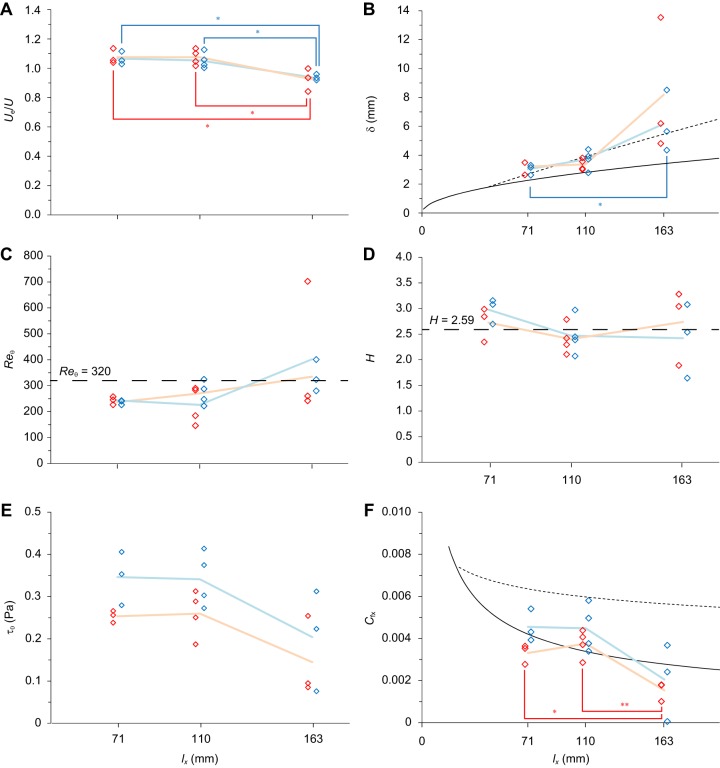
**Boundary layer-related parameters.** (A) The ratio of the near-field flow velocity to the freestream velocity, *U*_e_/*U*; (B) boundary layer thickness, δ; (C) the momentum thickness Reynolds number, *Re*_θ_; (D) shape factor, *H*; (E) local shear stress, τ_0_; and (F) skin friction coefficient, *C*_fx_, at three regions of the fish surface [pectoral region, arc length (*l_x_*) =71 mm; pelvic region, *l_x_*=110 mm; and posterior region, *l_x_*=163 mm]. The plots compare these parameters between the convex motion phase (red diamonds indicate pectoral: *N*=3, pelvic: *N*=4, posterior: *N*=3) and the concave motion phase (blue diamonds indicate pectoral: *N*=3, pelvic: *N*=4, posterior: *N*=3). The mean values in each of the three surface regions are connected by blue and orange lines for the concave and convex motion phases, respectively; *P<0.05 (for both red and blue); ** *P*<0.01. The solid and broken curves in panels B and F represent the estimates for a flat plate (semi-infinite in length) that were calculated theoretically assuming laminar and turbulent boundary layers, respectively.

The time average of the maximum tangential flow velocity (*U*_e_) out of the edge of the boundary layer [surface-normal distance, where tangential flow velocity (*u*) became 99% of freestream velocity (*U*)] that was normalized by the freestream velocity (*U*_e_/*U*) reached a maximum value of 1.08±0.05 (*N*=3) in the pectoral surface region (arc length measured from the rostrum, *l_x_*=71±8 mm, *N*=3) when the fish's surface was moving towards the freestream flow (convex motion phase) and 1.07±0.05 (*N*=3) in the same surface region when the fish's surface was retreating from the freestream flow (concave motion phase) (Fig. 1A). The mean *U*_e_/*U* ratio decreased posteriorly, and in particular, the mean value in the posterior surface region (*l_x_*=163±22 mm, *N*=3) became significantly smaller than those in the anterior surface regions in the same motion phase (one-way ANOVA, *P*<0.05, followed by a Tukey's *post hoc* HSD multiple comparison test, *P*<0.05). It is important to note that the *U*_e_/*U* ratio in the posterior surface region was less than 1.0 in both the convex and concave motion phases. As a result, the boundary layer thickness (δ), which is defined as a surface-normal distance where the *u* becomes 99% of the freestream velocity: δ=*y*(99%*U*), was replaced by a surface-normal distance where the *u* (<0.37 m s^−1^) became the maximum in the PIV flow field in comparison with published data. The time averages of the δ (Fig. 1B) in the pectoral and pelvic regions (*l_x_*=110±13 mm, *N*=4) are distributed around the estimates on the basis of turbulent boundary layer theory, the one seventh power law (Eqn 13 in Yanase and Saarenrinne, 2015). The empirical δ generally increased posteriorly and inflated in the posterior surface region. In particular, δ=6.17±2.13 mm (*N*=3) in the posterior surface region in the concave motion phase was significantly greater than δ=3.02±0.36 mm (*N*=3) in the pectoral surface region in the same motion phase (one-way ANOVA, *P*<0.01, followed by a Tukey's *post hoc* HSD multiple comparison test, *P*<0.05).

The time average of the Reynolds number (*Re*_θ_, Fig. 1C) based on momentum thickness (θ), which is the distance that is perpendicular to the fish surface through which the boundary layer momentum flows at freestream velocity, and *U*_e_ as a characteristic length scale in the pectoral and pelvic regions, was less than 320 in both the convex and concave motion phases. Preston (1958) proposed a *Re*_θ_ of 320, above which the boundary layer flow is likely to be fully turbulent. However, the *Re*_θ_ in the posterior region exceeded 320 and fluctuated widely. The displacement thickness (δ*) represents a virtual distance by which the fish's surface must be displaced outwards to yield the same flow rate as an inviscid flow at freestream velocity. The ratio of δ* to the θ, which is known as the shape factor (*H*, Fig. 1D), is used to evaluate unknown boundary layers for their proximity to a laminar or turbulent boundary layer profile. The empirical *H* in the pectoral and pelvic regions is distributed around *H*=2.59, which is the theoretical value of the laminar boundary layer (Schlichting, 1979).

The time average of the wall shear stress (τ_0_, Fig. 1E) in the convex motion phase was less than that of the concave motion phase in each of the three surface regions. The time averages of the skin friction coefficient (*C*_fx_, Fig. 1F) that were measured over the entire surface regions were less than those estimated on the basis of turbulent boundary layer theory, the one seventh power law (Eqn 12 in Yanase and Saarenrinne, 2015). This power law gives a good general description of the shape of the turbulent mean velocity profile in moderate, favourable pressure gradient regime flows. The *C*_fx_ in the pectoral and pelvic surface regions was distributed around the estimates for a laminar flat plate boundary layer based on the Blasius solution. The *C*_fx_=0.0015±0.0005 (*N*=3) of the posterior surface region in the convex motion phase was significantly lower than the *C*_fx_=0.0033±0.0005 (*N*=3) of the pectoral surface region and the *C*_fx_=0.0038±0.0007 (*N*=4) of the pelvic surface region in the same motion phase (one-way ANOVA, *P*<0.01, followed by a Tukey's *post hoc* HSD multiple comparison test, pectoral versus posterior: *P*<0.05, pelvic versus posterior: *P*<0.01).

### Swimming kinematics and the unsteady boundary layer profile

**Fig. 2.**
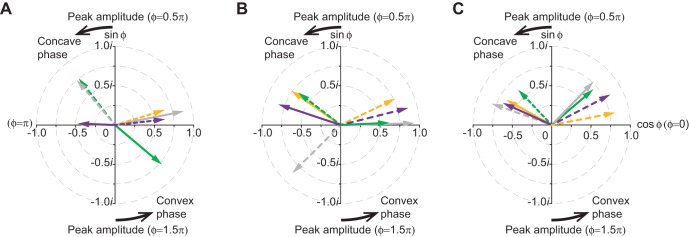
**The relationship between boundary layer-related parameters and the phase (φ) of the surface's movement.** The vertical displacement of the oscillatory fish surface is modelled with a sine wave. The peak displacements of the convex and concave surfaces correspond to φ*=*0.5π and φ=1.5π, respectively. Phase (φ) of the surface movement, at which the maximum value (solid arrows) and the minimum value (broken arrows) was recorded in (A) the pectoral region [arc length (*l_x_*)=71 mm], (B) the pelvic region (*l_x_*=110 mm), and (C) the posterior region (*l_x_*=163 mm), is indicated by the azimuth projected on a complex plane. The phase vectors for the *U*_e_/*U* ratio, the boundary layer thickness (δ), the wall shear stress (τ_0_), and the local friction coefficient (*C*_fx_) are indicated in grey, green, purple and orange, respectively. Time and phase increase in an anticlockwise direction. At φ=0 and π, the fish surface passed the mid-point of the time sequence in the convex motion and the concave motion phases, respectively. The longer the vector length is, the more oriented the phase vector is to the direction (one-sidedness).

Although the distribution waves of the *U*_e_/*U* ratio, δ, τ_0_ and *C*_fx_ in each of the three surface regions have the same frequency as the undulatory body wave, they maintained a constant phase difference (i.e. out-of-phase waves). The maximum and minimum peaks of these parameters are indicated by the azimuth that is projected onto the complex plane (*Re*^φ*i*^, where *R* is the vector length) in Fig. 2. The maximum τ_0_ occurred at 3.10±1.02 rad (*N*=3) in the pectoral surface region, 2.82±0.39 rad (*N*=4) in the pelvic surface region, and 2.72±0.27 rad (*N*=3) in the posterior surface region. In all cases, the maximum value occurred immediately before the mid-point (φ=π) of the time sequence in the concave motion phase. The minimum τ_0_ occurred at 0.11±0.66 rad (*N*=3) in the pectoral surface region, 0.25±0.50 rad (*N*=4) in the pelvic surface region, and 0.48±0.96 rad (*N*=3) in the posterior surface region. In each case, the minimum occurred immediately after the mid-point (φ=0) of the time sequence in the convex motion phase. The maximum and minimum τ_0_ in the phase plots (Fig. 2) revealed the phase-recessive and -progressive distributions in the stream wise direction in relation to the phase of the body travelling wave, respectively. The peak *C*_fx_ was almost in phase with the peak τ_0_. The peak δ and *U*_e_/*U* ratio were roughly π out of phase with the peak τ_0_ and *C*_fx_. However, except for τ_0,_ no regular stream wise phase-shift was found in the distribution wave of the other parameters.

**Fig. 3.**
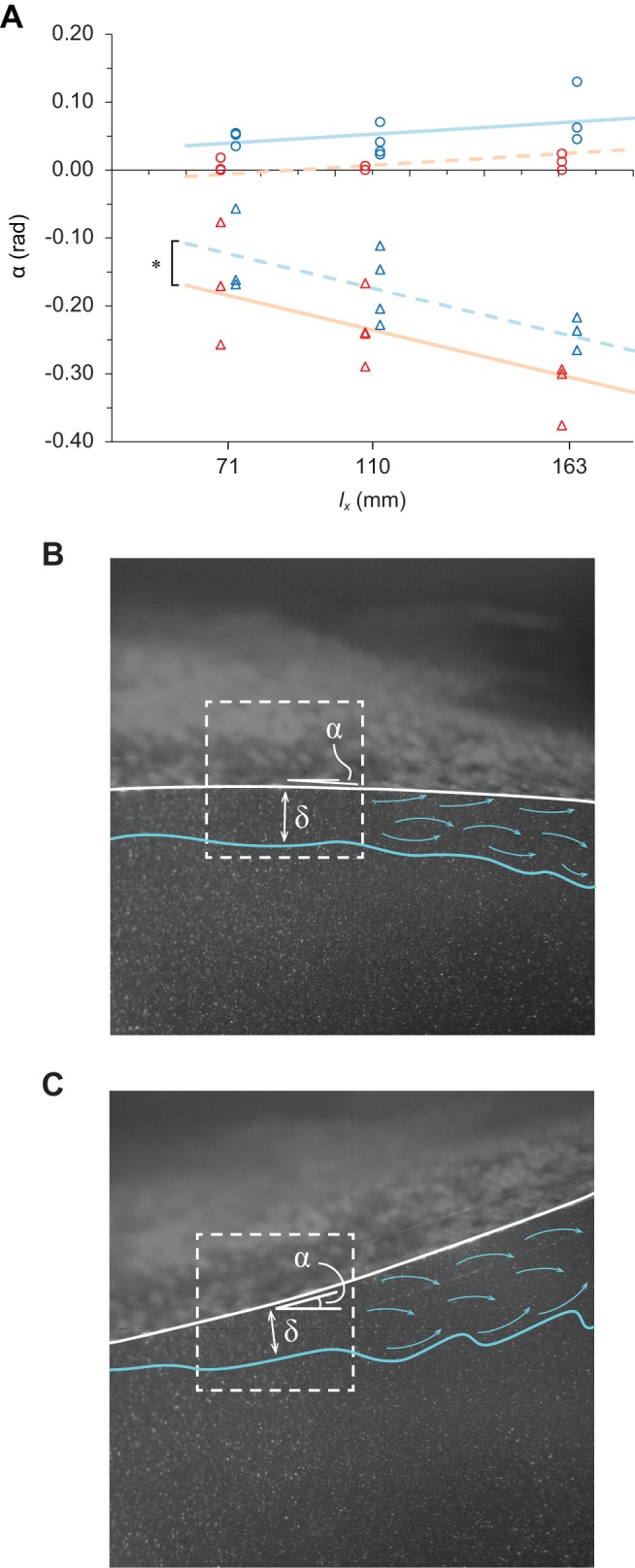
**Angle of peak incidence (α) for the propulsive fish surface relative to the freestream flow.** (A) The plots compare the α in the pectoral surface region [arc length, *l_x_*=71 mm; convex: positive peak, red circle (*N*=2) and negative peak, red triangle (*N*=3); concave: positive peak, blue circle (*N*=3) and negative peak, blue triangle (*N*=3)], the pelvic surface region [*l_x_*=110 mm; convex: positive peak, red circle (*N*=1) and negative maximum, red triangle (*N*=4); concave: positive peak, blue circle (*N*=4) and negative peak, blue triangle (*N*=4)], and posterior surface region [*l_x_*=163 mm; convex: positive peak, red circle (*N*=2) and negative peak, red triangle (*N*=3); concave: positive peak, blue circle (*N*=3) and negative peak, blue triangle (*N*=3)]. The solid and broken blue and orange lines represent the ANCOVA regressions for the concave and convex motion phases, respectively. *P<0.05. The images with the 45×45-mm^2^ area (B) and (C) show examples of the PIV flow field and the fish's body in the posterior region during the concave motion and convex motion sequences, respectively. In the images, the fish's surface reached a positive and negative peak incidence relative to the freestream flow. The blue lines and arrows show conceptual illustrations of the edge of the boundary layer with a thickness of δ and turbulent flows within the boundary layer. The broken-line square shows the 15×15 mm^2^ area that is observed by the boundary-layer camera.

The angle of incidence of the propulsive fish surface relative to the freestream flow generally reached a negative peak (α, Fig. 3A) immediately before the mid-point (φ=0) in the convex motion sequence. In the later phase of the convex motion sequence the α became positive (Fig. 3B). However, in some PIV trials of the pectoral and pelvic surface regions, the fish surface was consistently inclined at a negative angle of incidence relative to the freestream flow throughout the cycles of the transverse surface motion. There was also a negative incidence in the later phase of the concave motion sequence, but this was always of less magnitude than the negative peak α that was observed in the convex motion phase over the entire surface regions (ANCOVA, *F*_1,17_=6.23, *P*<0.05). The negative peak α increased in magnitude in line with an increasing *l_x_* and measured −0.323±0.083 rad (*N*=3) in the posterior surface region (Fig. 3C).

The body-wave velocity (*c*) was determined on the basis of the time shift of the peak lateral excursion of the fish's surface (out-of-phase standing waves) between two reference points, which were located a known distance apart in a 45×45 mm^2^ view field (approximately). The ratio of *c* to the free stream velocity (*U*) was a value that was, in general, close to 1.2 in both the pectoral (1.24±0.07, *N*=3) and pelvic regions (1.24±0.14, *N*=4). However, the *c*/*U* ratio in the posterior surface region was slightly greater (1.33±0.02, *N*=3).

**Fig. 4.**
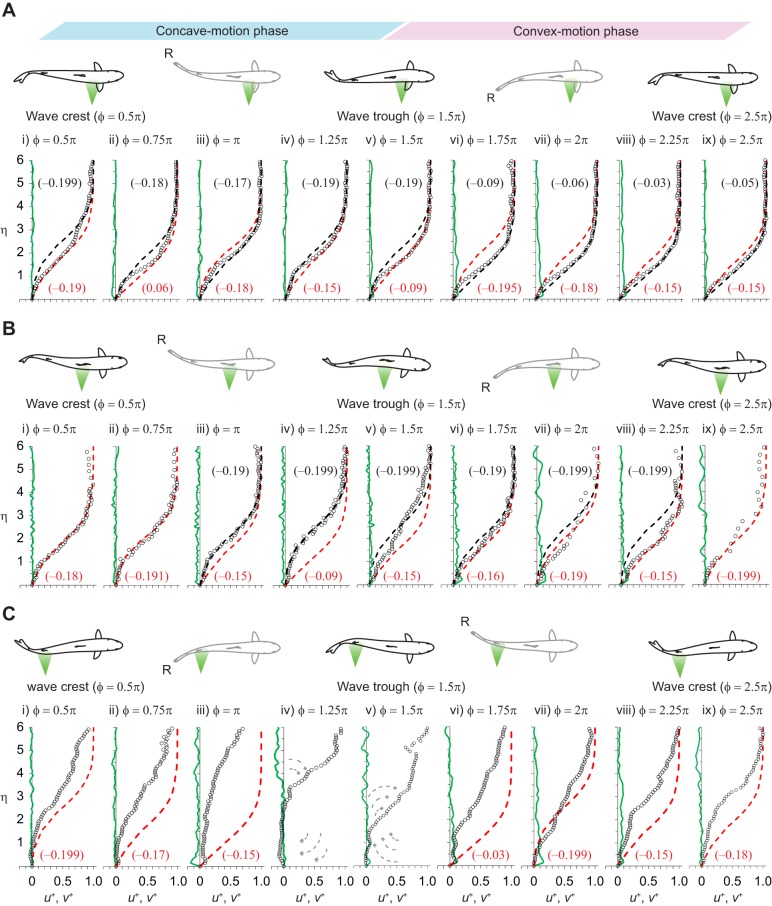
**Representatives of unsteady boundary-layer laminarization.** (A) The pectoral and (B) pelvic surface regions, and (C) inflected boundary layer in the posterior surface region in a single cycle of transverse surface motion. The boundary layer profiles in each of the three surface regions are approximated by the Falkner–Skan equation for a laminar boundary layer with different β values. The data plots show the vertical distribution of the normalized tangential velocity (*u*^+^: black circles) and normal velocity (*v*^+^: green line) over the fish surface. The cycle begins with the onset of the concave motion sequence (phase angle, φ=0.5π). The propulsive fish surface then reaches the wave trough (peak amplitude) at the end of the concave motion sequence (phase angle, φ=1.5π) and returns to the initial peak amplitude position (wave crest) at the end of the convex motion sequence (phase angle, φ=0.5π or 2.5π). The time series variation in the vertical gradient of the *u*^+^ velocity and *v*^+^ velocity within a single cycle is indicated from left to right with an increment of a phase angle of 0.25π. The grey broken arrows in panel C represent the separated shear flow in the form of a clockwise vortex. The red broken curve line represents the Falkner–Skan profile with a given β value that best approximated the initial rise of the velocity gradient at the fish surface [*f* '(0)]. The black broken curve line represents the Falkner–Skan profile with a different β value that could reasonably approximate the trend of the *u*^+^ velocity distribution in the intermediate layers or those that are closer to the normal edge of the boundary layer. The number in parentheses represents the β value. The β value for the corresponding Falkner–Skan profile is indicated in the parentheses by the same colour code (black or red) as the curve line. The fish illustrations describe the undulatory kinematics at important reference positions: at the outset and end of the concave motion sequence (φ=0.5π and 1.5π, respectively), the end of the convex motion sequence (φ=2.5π), and the reversal of the tail tip motion direction (denoted by R), which occurs between φ=0.5π−1.5π and between φ=1.5π−2.5π. The PIV flow field of interest is highlighted in light green, which is suggestive of a laser sheet.

Fig. 4 shows an example of the unsteady boundary layer profiles during a single cycle of transverse surface motion. It represents the general characteristics of the boundary layer over an undulatory fish surface, where the surface-normal distance, *y*, is normalized by *l_x_Re_x_*^−0.5^ (cf. Eqn 6), where *Re_x_* is the local Reynolds number that is based on the *U*_e_ and *l_x_*, and denoted by η. The tangential and normal components of the flow velocity are normalized by *U*_e_ and denoted by *u*^+^ and *v*^+^, respectively. The *u*^+^ velocity reached 1.0 around η=5 in the pectoral and pelvic surface regions. The vertical distributions of the *u*^+^ velocity in these surface regions were well approximated by the Falkner−Skan equation with a different parameter β, if separately analysed for the layers that are close to the fish's surface (red broken curve in Fig. 4A,B) and the intermediate layers or those that are closer to the *y*(*U*_e_) height (black broken curve in Fig. 4A,B). The parameter β can be interpreted geometrically as an angle of a reclining surface (βπ/2). However, the shape of the boundary layer profile in the pelvic surface region was somewhat destabilized from the beginning to the final phase of the convex motion sequence (Fig. 4B). In this phase of the convex motion sequence, the *u*^+^ velocities in the intermediate layers were slower than the theoretical limit of the *u*^+^ velocity to the boundary layer attachment (β=−0.199; e.g. φ=1.5π and 2π in Fig. 4B). The *v*^+^ velocities in the layers close to the surface of the fish were negative (suction flow) in the concave motion phase and positive (injection flow) in the convex motion phase. A complete separation of the boundary layer was observed in the posterior surface region during the convex motion sequence (φ=1.5π in Fig. 4C).

## DISCUSSION

### Boundary layer laminarization

The general characteristics of the phase of the maximum and minimum peak of the *U*_e_/*U* ratio, δ, τ_0_ and *C*_fx_ were the same as those in the turbulent boundary layers that were observed during low-speed swimming (Yanase and Saarenrinne, 2015) and contradicted the results of Anderson et al. (2001). In particular, the distribution wave of the *C*_fx_ (Fig. 2) suggests that the qualitative characteristics of *C*_fx_ agree with time-dependent simulations of skin friction at comparable positions over the surface of a moving flat plate, emulating aquatic animal locomotion (Ehrenstein and Eloy, 2013). However, it is somewhat questionable whether fluid behaviour around swimming fish could be simulated completely by computational fluid-dynamics software without considering the fluid-structure interactions between internal muscle, body stiffness or relaxation (Tytell et al., 2010), and perhaps the effect of mucus concentration in the layer immediately next to the fish's surface (i.e. the viscous sublayer).

The boundary layers of rainbow trout in the pectoral and pelvic surface regions were identified as being in a laminar flow regime when the fish was swimming at 1.6* L* s^−1^. As previously demonstrated in experiments using a mechanical model (Taneda and Tomonari, 1974; Kunze and Brücker, 2011), for the rainbow trout the ratio of the propulsive body-wave velocity (*c*) to the freestream velocity (*U*) was 1.2 for the reversion from a turbulent to a laminar flow regime in the boundary layer. This conclusion is based on the following measurements and the shape of the tangential velocity (*u^+^*) distribution in the boundary layers. The time average of the momentum-thickness Reynolds numbers (*Re*_θ_) in the pectoral and pelvic surface regions was less than 320, which is the threshold value for laminar-to-turbulent transition proposed by Preston (1958). The Falkner−Skan equation with a negative β closely approximated the distribution of the *u*^+^ velocity in the boundary layer of these regions. The negative β denotes the pressure increase in the direction of the fluid flow (adverse pressure gradient). The adverse pressure gradient tends to decelerate the flow in the boundary layer relative to its velocity in the absence of a pressure gradient (Schlichting, 1979). The increase in the thickness of the laminar boundary layer over the fish's surface (δ, Fig. 1B) is probably due to this pressure force being directed upstream.

### Boundary layer control for drag reduction

The present study examined the boundary layer of rainbow trout that were swimming at a speed of 1.6* L* s^−1^ under the assumption that this speed would be more energy-efficient for this species than the 1.0* L* s^−1^ swimming speed in our previous experiment (Yanase and Saarenrinne, 2015). When salmonids undergo tests on prolonged swimming, the critical swimming speed (*U*_crit_) test (Brett, 1964), they switch progressively from aerobic to anaerobic propulsion. In the *U*_crit_ test, a fish species of a certain length is subjected to increasing flow velocities in a series of steps maintained for fixed time periods until the fish is unable to swim in the flume due to fatigue. The energy expenditure of a rainbow trout moving one unit of body mass for one unit of distance decreased proportionally as the swimming speed increased from the lowest swimming speed, such as 1.0* L* s^−1^, to the least-cost swimming speed. The relationship between cost of transport (COT) and the swimming speed is characterized by a U-shaped curve (Yanase et al., 2012). Teulier et al. (2013) reported that the least-cost swimming speed occurred between 2.0* L* s^−1^ and 2.4* L* s^−1^ at 13°C. Therefore, the 1.6* L* s^−1^ swimming speed tested in the present study can be regarded as an energetically more favourable swimming speed for rainbow trout than 1.0* L* s^−1^. Given these considerations, the boundary layer laminarization of rainbow trout in turbulent flows observed in the present study should be part of the energy-efficient swimming strategies of this species in a riverine environment, where large and small scale vortices are embedded. This point will be discussed further in the following sections.

A laminar boundary layer creates less skin friction than a turbulent boundary layer. However, a laminar boundary layer can withstand only a small adverse pressure gradient before separating. Given that the shape factor (*H*) is an indicator of a pressure gradient, and hence of a separation tendency, it is known that flow separation is likely to occur around *H*=3.5 for a laminar boundary layer (Schlichting, 1979). The *H* for the boundary layer profile in the pectoral and pelvic surface regions, which was as great as 4.0, appeared during a cycle of the transverse surface motion. However, no boundary layer separation was observed in these surface regions. The flow profiles at the onset of the concave motion phase in the pectoral surface region in Fig. 4A (φ=0.5π) and at the onset of the convex motion phase in the pelvic surface region in Fig. 4B (φ=1.5π) were two extremes. The *u*^+^ velocities in the intermediate layers or in the layers close to the nominal edge of the boundary layer *y*(*u* =*U*_e_) of these profiles were slower than the predicted velocities from the Falkner−Skan equation with β=−0.199 (*m*=−0.091). This describes the onset of boundary layer separation and vanishing wall shear stress. However, the experimental observations were not in agreement with the most likely consequence at the boundary, in which there would have been boundary separation. The cause of such a flow profile deformation without separation can be explained partly by the physical impact caused by the reversal of the surface motion direction at φ=0.5π and 1.5π. During the rest of the convex motion sequence in the pelvic surface region (φ=1.75π−2.25π or 0.25π, Fig. 4B), the magnitude of the adverse pressure gradient in the intermediate layers was great enough to cause the onset of separation (β=−0.199). We attributed the avoidance of flow separation during this motion sequence to the direct acceleration of the water particles in the layers close to the boundary (i.e. the viscous sublayer) resulting from the normal force of the convex surface. Another possibility is that the effect of non-Newtonian fluid dynamics, known as ‘shear thinning’, decreases the apparent viscosity of the fluid as the shear rate increases (Ryder and Yeomans, 2006). On the basis of this assumption, the acceleration of the *u*^+^ velocity in the layers that are close to the boundary can be interpreted as indicating that the high molecular weight polymer chain of the mucus, which would be concentrated in the viscous sublayer, was stretched in a downstream direction along the fish surface by the interaction of the upper layers, thereby causing the Reynolds stress. Indeed, the mucus secretion from the relatively anterior surface of the fish was confirmed in the sequence images.

The greater magnitude of the negative peak incidence than the positive peak incidence (Fig. 3A) is probably because of the body shape of rainbow trout, which tapers posteriorly to the pectoral surface region near the tail. Indeed, as shown in Fig. 3C, the propulsive fish surface faced toward the tail in the majority of all the surface motion sequences, during which the flow area within the boundary layer increased considerably (i.e. flow diffusion) in comparison to the case of a flat plate having the same wetted surface area at a zero angle of incidence. These findings suggest that the boundary layer in the pectoral to posterior surface regions developed in a decelerating incident flow due to the increased stream-wise pressure gradient (the Bernoulli's principle). In addition, the stream-wise decrease in the *U*_e_/*U* ratio (Fig. 1A) can be interpreted as evidence that the boundary layer flow, which had presumably been accelerated to the maximum before reaching the pectoral surface region, was in a decelerating phase toward the tail. The increased adverse pressure gradient must have caused a significant loss in momentum flux within the boundary layer, particularly in the posterior surface region, during the convex motion sequence where the negative peak incidence (α) was −0.323 rad (Fig. 3A,C). The Falkner−Skan equation determines β as −0.199 (corresponding to −0.313 rad), which is the critical angle of incidence where boundary layer separation occurs. Thus, boundary layer separation was theoretically possible in the posterior surface region during the convex motion sequence. A sharp increase in the magnitude of the negative peak incidence for the convex surface was found in the posterior surface region (Fig. 3A). This may reflect the greater contribution to thrust generation of the fish surface upstream of the trailing edge in a subcarangiform swimmer, e.g. rainbow trout, than that of a carangiform swimmer (Webb, 1975), such as the scup tested by Anderson et al. (2001). Therefore, it is no surprise that the boundary layer separated from the posterior fish surface and not from the trailing edge. However, the occurrence of the separation was probably delayed in phase by the effect of wall suction. This is suggested by the negative *v*^+^ velocities (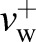) in the layer close to the boundary (φ=0.5π−π, Fig. 4C). More importantly, the negative *v*^+^ velocities in the layers that are close to the outer edge (η^+^≈5) at φ=1.25π in Fig. 4C suggest that the momentum transfer in the detached shear layer could be enhanced by the normal mass flux from free-streaming into the detached shear layer. Therefore, the boundary layer separation that was found in the final phase of the concave motion phase (φ=1.25π, Fig. 4C) was still marginal. However, the positive 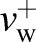 and minimal net mass flux from free-streaming (nearly zero *v*^+^ around η=5) at the onset of the convex motion phase in the same surface region (φ=1.5π, Fig. 4C) suggest that the separated shear flow in the form of a vortex with clockwise rotation (relative to the fish surface of interest) was shed in the freestream flow. The vortex with clockwise rotation would arrive at the trailing edge (tail tip) before the tail tip intersected the amplitude midline (φ=0) in the convex motion phase. Thus, a reverse Kármán vortex street (Müller, 2003) could be developed in the downstream wake.

Based on Webb's measurements (Webb, 1971), the drag experienced by rainbow trout swimming at sustained speeds exceeded that expected for a flat plate of the same wetted surface area by a mean factor of 3.03. The *C*_fx_ of swimming rainbow trout in the pectoral surface region during the concave motion sequence and in the pelvic surface region during the convex motion sequence exceeded the estimates for a laminar flat plate boundary layer. However, as Fig. 1F shows, the difference may be cancelled out entirely in one cycle of the surface movements by the skin friction reduction that occurred at the same time on the contralateral side of the fish. Therefore, it is plausible that rainbow trout that were swimming at 1.6* L* s^−1^ would experience as low a frictional drag as does a laminar boundary layer over a flat plate of the same wetted surface area. This means that the Bone–Lighthill boundary layer thinning hypothesis, which is that the undulatory motions of swimming fish cause a large increase in their friction drag because of the boundary layer compression, was not supported. As long as the attachment of the boundary layer to the fish surface upstream of the posterior surface region was maintained, no extreme increase in pressure drag is likely to occur. This being so, the challenge is to explain the cause of the large increase in drag measured by Webb (1971). We believe that the most likely cause is the transverse separation of the boundary layer at the ventral and dorsal edges of the fish's lateral surfaces and the leading edge (i.e. cross-flow separation). Unfortunately, the current study was limited to two-dimensional PIV measurements in a horizontal plane along the length of a steadily swimming fish. How the fish is able to address such a three-dimensional effect is not completely understood. With increasing swimming speed, this effect could potentially be accompanied by a large increase in the drag power, which is proportional to the third power of the velocity of the oscillatory fish surface. Meanwhile, the leading surface in the posterior half of the body, which faced upstream in the later phase of the convex motion, has to move more quickly against the flow passing the swimming fish in order to produce greater thrust.

In summary, we have confirmed the boundary layer laminarization of rainbow trout swimming at a sustained speed of 1.6* L *s^−1^, which was suggestive of the strategy for energy-efficient locomotion in the turbulent flow environment that this species inhabits. The boundary layer over the trailing surface in the middle of the fish's body (pectoral and pelvic regions) was generally laminar, whereas it was more or less destabilized over the leading surface. The pressure gradient along the curved surface of the fish tended to be adverse during the entire cycle of transverse surface movement. The ratio of the body-wave velocity (*c*) to the swimming speed (*U*) was in the order of 1.2 when the boundary layer became laminar. The *c*/*U* ratio of 1.2 was consistent with the condition of the boundary-layer laminarization that had been confirmed experimentally using a mechanical model of a flexible flat plate that emulates fish-like locomotion and computational fluid dynamic (CFD)-based numerical simulations.

## MATERIALS AND METHODS

### Fish

The rainbow trout were obtained from Pohtiolampi Osprey Centre (Kangasala, Finland), a fish farm. The fish were held indoors in a 300 l aquarium at the Flow Research Laboratory of Tampere University of Technology, Finland. The water in the holding tank was sufficiently oxygenized and maintained in an appropriate condition while being recirculated through a filtration system. The Act on the Use of Animals for Experimental Purposes (62/2006) (Suomen national animal welfare law) defines that experiments using farmed fish for production purposes are not animal experiments. Therefore, we confirmed through discussions with the authorities that our experiment was not subject to the law.

### Experimental flow system

**Fig. 5.**
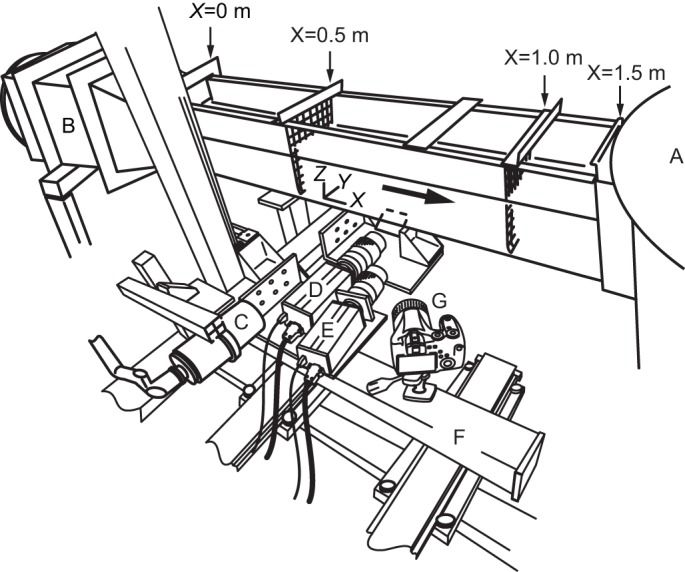
**An overview of the experimental set-up.** The flow drawn from a settling tank (A) was delivered to the open channel after the flow area was reduced at a contraction (B) and directly upstream of the open channel (*X*=0.5−1.0 m). The arrow represents the direction of a seeded flow. PIV images were acquired in a horizontal mid-plane of the fish illuminated from the side using an Nd:YLF-pulsed laser through light sheet optics (C) and imaged from the bottom by a mirror using two high-speed cameras, which are designated as the boundary-layer camera (D) and the near-field camera (E). These cameras were mounted on a motor-driven slider (F). A lateral view of the swimming behaviour of the fish was monitored using a high-speed camera (G).

The experiment was carried out using Tampere University of Technology's experimental flow system (Fig. 5). Unidirectional flow at 20°C was induced in the 1.5 m open channel of the recirculating flow system. Assuming that the turbulence in the freestream was isotropic, the turbulence intensity (*T*) is defined as *T*=*u*_rms_/*U*, where *u*_rms_ is the root mean square of the fluctuating velocity component in the freestream direction (Schlichting, 1979). The *T* in the freestream (*U*=0.370±0.021 m s^−1^) that was measured using the PIV technique over 16 points at different depths (0.04−0.08 m from the bottom) in a 0.21×0.24×0.50 m (width×height×length) test section (without fish) was 5.6±1.7%. The volumetric flow rate did not change during the experimental trials. Consequently, the depth of water in the test section was maintained at 0.11 m. The PIV measurements were conducted when the fish displayed station-holding behaviour at 1.60±0.09* L* s^−1^ (*N*=6) relative to the open channel. As the cross-sectional area of the fish was sufficiently small in comparison to the cross-sectional flow area of the open channel, there was no need to correct the flow velocity because of the solid blocking effect (Bell and Terhune, 1970). For more detail on the experimental flow system, refer to Yanase and Saarenrinne (2015).

### PIV image acquisition

**Fig. 6.**
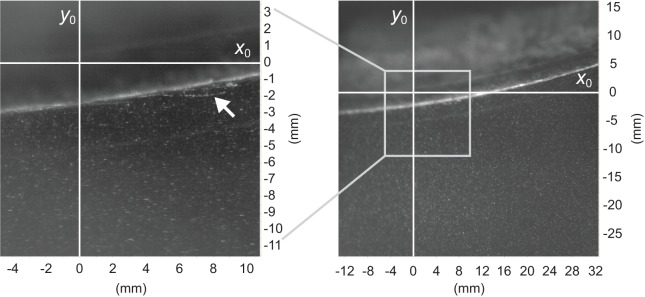
**PIV images acquired from two cameras and a definition of the 2D-coordinate system that the two cameras shared.** Boundary-layer camera: left image and near-field camera: right image. This image pair was acquired at an arc length (*l*_x_) of 80 mm measured from the rostrum. The area in the white square in the right image corresponds to the image on the left. The positive *x*_0_ direction represents the free-flow direction that is associated with tangential velocity (*u*) relative to the fish's surface. The transverse surface movements in the positive *y*_0_ direction and negative *y*_0_ direction are described in the text as concave motion and convex motion, respectively. The white arrow in the left image indicates the plume of mucus that was secreted from the fish surface.

A rainbow trout was constrained to swim in the test section. After an hour of acclimation to the experimental environment by the fish, PIV images were acquired from the bottom of the test section using a mirror angled at 45° and two high-speed cameras (ImagerProHS, LaVision, Göttingen, Germany). A number of singly-exposed particle image pairs with a resolution of 1040×1024 pixels were recorded simultaneously by these cameras at 200 frames s^−1^. One camera, with a ∼15×15 mm^2^ field of view was used to image the boundary layer. This camera is referred to as the boundary-layer camera. The other camera has a ∼45×45 mm^2^ field of view and is referred to as the near-field camera. The cameras took two frames per image, during which their shutters remained open; this is also known as the ‘double frame’ mode. The flow field that was seeded with neutrally buoyant tracer particles was illuminated by a horizontal laser sheet with a pulse delay of 300 µs (Nd:YLF pulsed laser, ESI New Wave Division, Cambridge, UK). Small glass spheres of 10 μm diameter were selected as seeding particles (LaVision, Göttingen, Germany) to ensure an adequate tracer response to the particles in turbulent flow (Hadad and Gurka, 2013). The laser and both the cameras were synchronized with the pulse generated by a software-programmable timing unit (PTU-9, LaVision, Göttingen, Germany) under the control of DaVis software (ver 7.2, LaVision, Göttingen, Germany). Both the boundary-layer camera and the near-field camera completed PIV flow imaging with the use of a 105 mm macro lens (Sigma 105 mm f2.8D EX DG, Sigma, Tokyo, Japan). To acquire highly resolved particle image pairs of the boundary layer with as little image distortion as possible, a teleconverter extension ring (N-AFD 1.5× TLLEPLUS SHQ, Kenko, Tokyo, Japan) was used for the boundary-layer camera. To create the 2D-coordinate system that could be shared by the two cameras (Fig. 6), a tilt/shift lens adaptor was used for the near-field camera.

The boundary-layer camera and the near-field camera were mounted on a custom built motor-driven slider so that fine adjustments could be made to their positions using joysticks on the controller (Motionline, Lenord, Bauer & Co., Oberhausen, Germany). To describe the oscillatory motion of the fish's surface, we use the terms ‘convex’ and ‘concave’ depending on the direction of the transverse surface movement in the ∼15×15 mm^2^ field of view of the boundary-layer camera. A convex motion describes the situation when the section of the fish's surface in the field of view of the boundary-layer camera was directed in the negative *y*_0_ direction in Fig. 6. This was while the fish surface was moving towards the freestream flow. Concave motion describes the situation when the section of the fish's surface in view was directed in the positive *y*_0_ direction in Fig. 6. This was while the fish's surface was retreating from its extreme position after the convex motion sequence had been completed. To locate the position of the laser sheet on the fish's surface, the lateral view of the test section was displayed on the computer screen through a high-speed camera (EX-F1, Casio, Tokyo, Japan). Immediately after PIV flow imaging, the fish was humanely killed through the administration of an anaesthetic (emulsified solution of oil of cloves: five drops of 100% pure oil of cloves per four litres of water).

### Post-PIV analysis of boundary layer-related parameters

Each pair of PIV images was analysed using the PIV flow-imaging software, DaVis (ver 7.2, LaVision, Göttingen, Germany), based on a multi-pass interrogation algorithm, where the search window for peak correlation was reduced by three steps from an initial interrogation window size of 32×32 pixels to a final window size of 8×8 pixels or 6×6 pixels, while maintaining a 50% overlap. The magnification factors were 23 pixels (mm)^−1^ for the near-field camera and 65 pixels (mm)^−1^ for the boundary-layer camera. Therefore, velocity vectors were analysed at a maximum of 341×347 node points (118,329 vectors) equally spaced at approximately 0.05 mm (3 pixels) in the particle images from the boundary-layer camera.

Post-PIV analysis of the boundary layer-related parameters during 1−10 cycles of tail oscillation was conducted on 200−1000 high quality sequential image pairs using customised software written in Matlab (R2012b v.8.0.0.783, MathWorks, Natick, MA, USA). To eliminate any influence on the accuracy of the estimates caused by the boundary layers that developed near the side walls and the floor of the flow channel, sequential image pairs acquired between 0.04 and 0.08 m above the bottom of the test section and more than 0.04 m from the side walls of the test section were used in the analysis. After the targeted PIV image was optimized, using the Gaussian or average filter to eliminate the noise of the image, the fish's surface was separated from the background by the Sobel edge-detection operator in Matlab and approximated by a fourth degree polynomial function. The wall shear stress (τ_0_) was estimated from the linear slope of the velocity profiles in the immediate neighbourhood of the surface (Eqn 1 at *y*=0; Kähler et al., 2006). It was considered to be statistically reliable if this analysis was undertaken at 100 Hz. Determining the normal distance (*d*) from a particular data sampling point to the surface of the fish was treated as a problem of finding the shortest path between the point and curve. Tangential flow velocity (*u*) was defined as a velocity component that was parallel to the tangent line at the point on the surface where *d* was determined. Normal velocity (*v*) was defined as a component of the velocity normal to the tangent line.

When the distribution of tangential flow velocity within the boundary layer is considered, it is commonly assumed that the velocity profiles at different positions along the surface differ only by a scale factor in surface-normal distance. This type of boundary layer problem is expressed in the form of self-similar solutions of the boundary layer equations (i.e. a Blasius boundary layer). The boundary layer thickness (δ) is arbitrarily defined as the normal distance from the surface to the point where the tangential flow velocity, *u*, is 99% of the freestream velocity. To extend this analysis to more general geometries, it is assumed that the boundary-layer-edge velocity, *U*_e_(*l*_x_), satisfies the power law:
(2)
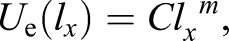
where *C* is a constant, and *l_x_* is the arc length measured on the fish's surface from the rostrum. When the similarity variable, η (=*y*δ^−1^), is defined, distribution of the non-dimensional velocity (*u*^+^=*u*/*U*_e_) in the boundary layer is derived as the solution of the ordinary differential equation, the so-called Falkner−Skan equation (Falkner and Skan, 1931):
(3)
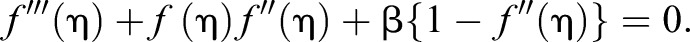
The coefficient β is defined by the relationship:
(4)
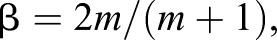
where *m* is the pressure-gradient parameter. The boundary conditions are determined to be *f* '(0)=0 (*u*=0), *f* (0)=0 (*v*=0), and *f* '(→∞)=1 (*u*=*U*e). The solution of Eqn 3 provides the closest approximation of the laminar boundary layer profile with acceleration or deceleration, and, thus, the pressure gradient. The case in which *m*<0, and, hence, −2<β<0, can be interpreted as the profile of flow over a reclining surface that makes an angle of βπ/2 with the freestream. A negative incidence corresponds to a decelerated flow along the surface with an adverse pressure gradient. The case of *m*>0, and hence, 0<β<2, can be interpreted as the profile of the flow past a sharp wedge of the βπ angle. A positive incidence corresponds to an accelerated flow along the wedge's surface with a favourable pressure gradient. The case of *m*=0, which gives zero flow acceleration, is a special case that corresponds to the Blasius boundary layer (Schlichting, 1979). Using the numerical solution (e.g. Howarth, 1938) for f'(*η*)=0.99 at *m*=0 and the original definition of the local friction coefficient, *C*fx=τ_0_/(0.5ρ*U*e^2^), where ρ is water density, the *C*fx and δ for the laminar boundary layer without streamwise pressure gradient are described as:
(5)
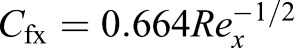
and
(6)
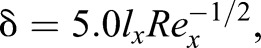
where *Re_x_* is the Reynolds number based on the arc length measured along the surface of the fish from the rostrum.

**Table 1.**
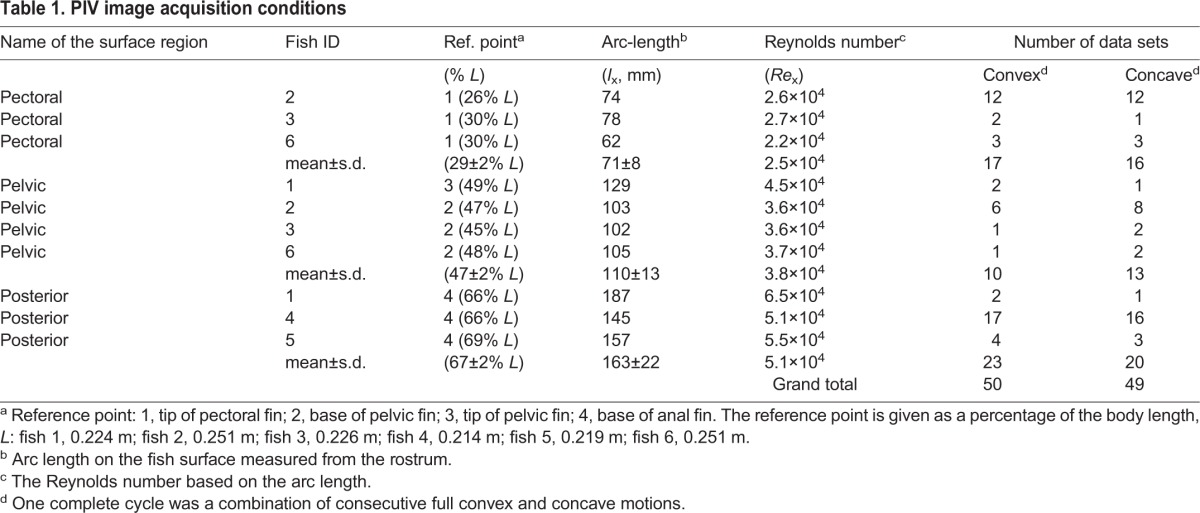
**PIV image acquisition conditions**

As mentioned earlier, the definition of the δ by 99% freestream velocity height from the fish's surface is arbitrary. This is especially true in a flow field that involves self-generated locomotor vortices and external vortices that are present in the flow environment. Therefore, we also used more meaningful measures to describe the boundary layer shape, the displacement thickness (δ*), and the momentum thickness (θ). These are defined as
(7)
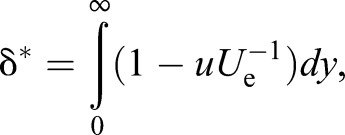
and
(8)
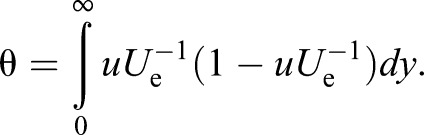
The definite integrals for Eqns 7 and 8 were approximated by adding the areas of rectangles that have a height of approximately 0.05−0.06 mm from the surface to the point where the tangential velocity (*u*) became the maximum (*U*_e_). The θ is a virtual surface-normal distance by which the boundary would be displaced to compensate for the momentum reduction of the flowing fluid due to the presence of the boundary layer. The Reynolds number, *Re*_θ_ (which was calculated with the local value of θ as a characteristic length scale and *U*_e_), was used to determine whether the boundary layer is laminar or turbulent. Preston (1958) proposed a *Re*_θ_ of 320, above which the boundary layer flow is likely to be fully turbulent. Assuming that an inviscid fluid flows along a surface, the solid surface would have to be displaced outwards by a distance of δ* to yield the same flow rate as an inviscid flow. The ratio of δ* to θ is thus
(9)
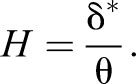
This is called the shape factor, which acts as an indicator of the pressure gradient and, hence, of the separation tendency (Schlichting, 1979). The higher the value of *H*, the greater the pressure that is directed in the upstream direction (i.e. adverse pressure gradient). It is known that the *H* value is 1.3 when the boundary layer profile follows the one seventh power law of turbulent velocity distribution. However, when *H* is 2.59, the boundary layer profile follows the Blasius profile for a laminar boundary layer without a streamwise pressure gradient (Schlichting, 1979). Therefore, the *H* is also used to evaluate any unknown boundary layers for their similarity to a laminar or turbulent boundary layer profile.

### Statistics

The basic statistics of the boundary-layer-related parameters (i.e. the *U*_e_/*U* ratio, δ, τ_0_, and C_fx_) and the pairwise comparisons of these parameters were performed using the Analysis ToolPak of Microsoft Excel 2010. The statistical program R (ver. 2.12.2, R Development Core Team, Vienna, Austria) was used to run ANCOVA and multiple-group comparisons with *post hoc* analysis. The surface of the body of the rainbow trout [0.231±0.016 m *L* (mean±s.d.); *N*=6] was divided into three regions, as in the previous experiment (Yanase and Saarenrinne, 2015), based on which body part of the fish was used as a reference point to measure the arc length (*l_x_* ±s.d.) of the fish's surface from the rostrum (Table 1). Unless stated otherwise, the boundary layer-related parameters are presented as a mean±s.d. of all the means that were determined from different sub-samples within each fish for each of the three surface regions. The measurements that were collected while the fish was swimming were separately analysed, depending on the direction of the transverse surface movements (Fig. 6) after the previous experiment (Yanase and Saarenrinne, 2015).
